# Involving the health sector in the prevention and care of female genital mutilation: results from formative research in Guinea

**DOI:** 10.1186/s12978-022-01428-4

**Published:** 2022-07-08

**Authors:** Mamadou Dioulde Balde, Anne Marie Soumah, Aissatou Diallo, Alpha Oumar Sall, Vernon Mochache, Wisal Ahmed, Amadou Oury Toure, Ramata Diallo, Sadan Camara, Sarah O’Neill, Christina C. Pallitto

**Affiliations:** 1 Centre for Research on Reproductive Health in Guinea/Cellule de Recherche en Santé de la Reproduction en Guinée (CERREGUI), Conakry, BP 4880 Guinea; 2 École de Santé Publique and Faculté de Philosophie et de Sciences Sociales, Université Libre de Bruxelles, U.L.B., CP 177, 50 Avenue F.D. Roosevelt, 1050 Brussels, Belgium; 3 UNDP-UNFPA-UNICEF-WHO-World Bank Special Programme of Research, Development and Research Training in Human Reproduction (HRP), Department of Sexual and Reproductive Health and Research, World Health Organization, 20 Avenue Appia, 1211 Geneva, Switzerland

**Keywords:** Female genital mutilation, FGM medicalization, Health sector, Formative research, Guinea, Mutilation génitale féminine, Médicalisation des MGF, Secteur de la santé, Recherche formative, Guinée

## Abstract

**Background:**

Despite efforts to reduce the burden of female genital mutilation (FGM) in Guinea, the practice remains prevalent, and health care providers are increasingly being implicated in its medicalization. This formative study was conducted to understand the factors that facilitate or impede the health sector in providing FGM prevention and care services to inform the development of health sector-based interventions.

**Methods:**

Between April and May 2018, a mixed methods formative study was carried out using a rapid assessment methodology in three regions of Guinea—Faranah, Labe and Conakry. A structured questionnaire was completed by one hundred and fifty health care providers of different cadres and 37 semi-structured interviews were conducted with health care providers, women seeking services at public health clinics and key stakeholders, including health systems managers, heads of professional associations and schools of nursing, midwifery, and medicine as well as representatives of the Ministry of Health. Eleven focus group discussions were conducted with female and male community members.

**Results:**

This study revealed health systems factors, attitudinal factors held by health care providers, and other factors, that may not only promote FGM medicalization but also impede a comprehensive health sector response. Our findings confirm that there is currently no standardized pre-service training on how to assess, document and manage complications of FGM nor are there interventions to promote the prevention of the practice within the health sector. This research also demonstrates the deeply held beliefs of health care providers and community members that perpetuate this practice, and which need to be addressed as part of a health sector approach to FGM prevention.

**Conclusion:**

As integral members of FGM practicing communities, health care providers understand community beliefs and norms, making them potential change agents. The health sector can support them by incorporating FGM content into their clinical training, ensuring accountability to legal and policy standards, and promoting FGM abandonment as part of a multi-sectoral approach. The findings from this formative research have informed the development of a health sector intervention that is being field tested as part of a multi-country implementation research study in Guinea, Kenya, and Somalia.

## Plain language summary

Despite the implementation of various interventions to prevent female genital mutilation (FGM), it is still widely practiced in Guinea, and health care providers are increasingly being implicated in the practice. We conducted research in three regions of Guinea, namely, Faranah, Labe and Conakry, to understand factors that might be addressed to strengthen the role of the health sector in prevention and care of women and girls who have undergone FGM. Our findings highlight the need to strengthen the capacity of health care providers to be able to identify cases of FGM and manage complications. The study also highlights the importance of engaging health care providers in efforts to prevent FGM, which will require that any trainings include an opportunity to discuss their own values and beliefs around FGM so that they are better equipped to communicate with their clients and patients in a sensitive and non-judgmental manner, whether during consultation visits or community health outreach activities. The results of this research have informed the development of a health system strengthening intervention package for the prevention and care of FGM, which is being tested in Kenya, Somalia, and Guinea.

## Résumé

**Contexte:**

Malgré les efforts déployés pour réduire le fardeau des mutilations génitales féminines (MGF) en Guinée, cette pratique reste répandue et les prestataires de soins de santé sont de plus en plus impliqués dans sa médicalisation. Cette étude formative a été menée pour comprendre les facteurs qui facilitent ou empêchent le secteur de la santé de fournir des services de prévention et de soins des MGF afin de promouvoir le développement d'interventions basées sur le secteur de la santé.

**Méthodes:**

Entre avril et mai 2018, une étude formative à méthodes mixtes a été réalisée en utilisant une méthodologie d'évaluation dans trois régions de la Guinée: Faranah, Labe et Conakry. Un questionnaire structuré a été rempli par cent cinquante prestataires de soins de santé de différentes structures de santé et 37 entretiens semi-structurés ont été menés avec des prestataires de soins de santé, des femmes sollicitant des services dans des structures de santé publique et des parties prenantes clés, notamment des gestionnaires de systèmes de santé, des responsables d'associations professionnelles et d'écoles d'infirmières, de sages-femmes et de médecine, ainsi que des représentants du ministère de la Santé. Onze discussions de groupe ont été menées avec des membres féminins et masculins de la communauté.

**Résultats:**

Cette étude a révélé des facteurs liés aux systèmes de santé, des facteurs liés aux attitudes des prestataires de soins de santé, et d'autres facteurs, qui peuvent non seulement promouvoir la médicalisation des MGF mais aussi empêcher une réponse globale du secteur de la santé. Nos résultats confirment qu'il n'existe actuellement aucune formation initiale standardisée sur la manière d'évaluer, de documenter et de gérer les complications des MGF, ni aucune intervention visant à promouvoir la prévention de cette pratique dans le secteur de la santé. Cette recherche démontre également les croyances profondes des prestataires de soins de santé et des membres de la communauté qui perpétuent cette pratique, et qui doivent être abordées dans le cadre d'une approche du secteur de la santé pour la prévention des MGF.

**Conclusion:**

En tant que membres à part entière des communautés pratiquant les MGF, les prestataires de soins de santé comprennent les croyances et les normes de la communauté, ce qui fait d'eux des agents de changement potentiels. Le secteur de la santé peut les soutenir en intégrant le contenu des MGF dans leur formation sanitaire, en veillant à ce qu'ils respectent les normes juridiques et politiques et en promouvant l'abandon des MGF dans le cadre d'une approche multisectorielle. Les résultats de cette recherche formative ont permis de développer une intervention du secteur de la santé qui sera testée sur le terrain dans le cadre d'une étude de mise en œuvre multi-pays en Guinée, au Kenya et en Somalie.

## Background

The harmful practice of female genital mutilation (FGM) affects more than 200 million women and girls aged 15–49 years worldwide [1]. It involves the partial or total removal of external female genitalia or other injury to the female genitals for non-medical reasons. FGM has no health benefits, interferes with the body’s natural functions and is a violation of human rights [2, 3].

Guinea, located on the West African coast, has one of the highest FGM prevalence rates in the world with 95% of Guinean women aged 15–49 years reported to have undergone FGM [4]. The number of Guinean women and girls that have undergone FGM varies by ethnicity. The practice is nearly universal in some ethnic groups, while in others, such as the Toma and Guerze, the prevalence is 90% and 68.4%, respectively. According to the 2018 Guinea Demographic Health Survey (GDHS), of the Guinean women who underwent FGM, the majority (87%) reported having undergone the practice before turning 15 years old, mostly between ages five and nine years, 9% were unsure and 4% reported having undergone FGM at age 15 or older.

The most prevalent forms of FGM reported were Type I or Type II, in which flesh was cut and removed (58%). The prevalence of Type III, in which flesh is removed and the vaginal opening is sewn closed was 10%. Cutting without removal of flesh was reported by 11% of women, while 21% did not know which type they had undergone [4]. Additionally, of the FGM cases reported by mothers of girls aged 0–14 years old, 59% stated that the practice was performed by a traditional practitioner while 35% reported that it was performed by a health care provider, also known as FGM medicalization. The rate of FGM medicalization in this age group was twice as high as that in the 15–49-year age group, indicating an increasing trend.

This increasing trend in FGM medicalization in Guinea and elsewhere led to the development of the World Health Organization (WHO)’s Global Strategy to Stop Health Care Providers from Performing FGM [5]. This strategy has four pillars that guide countries in their actions against FGM medicalization including: (1) mobilizing funds and political will to address FGM; (2) strengthening knowledge and skills of health providers; (3) creating legislative and regulatory frameworks and (4) strengthening monitoring, evaluation, and accountability systems.

A multi-country literature review on what motivates health care providers to conduct FGM found that strong community demand, the influence of cultural norms and personal experiences within their communities of origin, perceived harm reduction when health care providers perform the practice themselves and personal financial incentives, were key drivers of FGM medicalization [6]. According to the 2018 GDHS, while many respondents indicated that health care providers were performing FGM, respondents did not know the cadre of health care provider nor whether they were trained and licensed providers. Regardless, the involvement of health care providers in performing FGM is concerning because it violates medical and professional ethics. It may also inadvertently perpetuate the practice by conferring a sense of legitimacy given the social status of health care providers in their communities.

Understanding the community and health sector perspectives on FGM medicalization is an important step in developing health sector approaches to address it. With adequate support and training, health care providers, as respected and influential community members, could play an important role in national efforts towards FGM abandonment in addition to providing appropriate care for FGM-related health complications [7]. There is, however, a dearth of evidence on what works to effectively involve them in FGM prevention and care efforts.

In this regard, formative research was carried out by the Centre for Research on Reproductive Health in Guinea—*Cellule de recherche en santé de la reproduction en Guinée (CERREGUI)* with the support of WHO and HRP during the months of April and May 2018, to inform the development of an intervention targeting health care providers to strengthen their role in FGM prevention and care by gaining a better understanding of the contextual situation regarding FGM and its medicalization in Guinea. The intervention package is being field tested in a multi-country implementation research study in Kenya, Guinea, and Somalia [8]. The specific objective of this paper is to discuss the factors that emerged from this formative research that facilitate or impede the health sector in its role in strengthening FGM prevention and care efforts.

## Methods

### Study design, population and sampling

This formative research employed a rapid assessment approach using mixed methods, to inform intervention development. Rapid assessments have been used for decades [9] to understand the contextual and systems level factors that underlie a particular issue as well as to consider the perceptions of key population groups when developing strategies that are contextually relevant. Rapid assessment approaches have also been used to address a range of health topics [10].

For this formative research, quantitative data collection included a questionnaire applied to a sample of 150 health care providers of different cadres including doctors, nurses, midwives, and auxiliary nurses. The quantitative survey was limited to Conakry, the capital city, because health care providers in Guinea are more centralized in the capital as opposed to other areas of the country where they are more sparsely distributed. This enabled data collectors to access enough health care providers to respond to the questionnaire in an efficient manner. Providers in Conakry were randomly selected from community health centers as well as larger hospitals based on a sampling framework using a probability of selection proportional to size.

For qualitative data collection, three groups were recruited for in-depth interviews (IDIs) and focus group discussions (FGDs). These included: (1) health care providers and management staff from health facilities, professional associations, or teaching institutions; (2) health facility users and (3) community members. These study participants were selected based on the different roles they play within the health sector to bring out various perspectives of health sector actors. Purposive sampling of potential respondents from these groups was used to capture a range of ethnic and cultural perspectives. Qualitative study respondents were sampled from three different regions namely, Faranah, Labé and Conakry, to provide geographic and ethnic representation and allow for urban and rural sampling that ensured a broader diversity of perspectives.

### Eligibility criteria

Participants were selected based on the respondent group they represent, their geographic location within the selected regions as described above and their availability and willingness to participate on the day of data collection.

### Study procedures

The quantitative aspect of the study focused on the knowledge and attitudes of health care providers towards FGM and its medicalization, and their practices regarding the care and treatment of women with FGM. Quantitative data collection was conducted by trained research assistants using paper-based questionnaires with manual recording of participants’ responses. The IDIs were used to understand health care providers’ attitudes and motivations around medicalization of FGM while FGDs were used to determine community members’ attitudes and perceptions. All qualitative interviews (FGDs and IDIs) took place in a private setting and were audio-recorded, lasting between 60 and 90 min. Given the sensitivity of the topics to be discussed, respondents were interviewed by interviewers of the same sex. After data collection, the audio recordings in local languages were translated and transcribed into French in preparation for analysis. Analysis was conducted on the French qualitative data. Selected quotations included in this paper were translated to English.

### Data analysis

Quantitative data were entered into the Census and Survey Processing System (CSPro) software (Version 7.6.2 census.gov/data/software/cspro.html) then transferred to International Business Machines (IBM) Corporation’s Statistical Package for the Social Sciences (SPSS) (IBM SPSS Statistics for Windows, Version 20.0. Armonk, New York, USA) for analysis by a trained data specialist in Guinea. Descriptive statistics as well as summary results of the knowledge, attitudes, and practices of the health providers were generated and have been published [11]. Bivariate analyses to compare response options among sub-groups of respondents based on selected socio-demographic characteristics were carried out and are presented in this paper.

Qualitative data were analyzed using a thematic analysis approach that informed the development of a conceptual framework regarding influences on health care provider knowledge, attitudes, and behaviors. This framework included three categories of drivers: (1) health system structural and functional factors around provision of services; (2) community factors driving demand for medicalized FGM and (3) individual level factors that shape attitudes and practices of health care providers. This conceptual framework was used to categorize both quantitative and qualitative results described in this paper.

### Ethical considerations

The protocol for this study was approved by the national ethical committee in Guinea (099/CNERS/17 November 30, 2017) and WHO’s Ethical Review Committee (ERC 0002962v3 20/11/2017). Written informed consent was obtained from the research participants prior to any data collection activities. The consent forms were translated into three local languages i.e., Soussou, Malinké and Pulaar.

## Results

Table 1 summarizes the socio-demographic characteristics of quantitative survey participants. A total of 150 questionnaires were completed by 15 (10%) obstetricians/gynecologists, 35 (23%) other doctors, 50 (33%) midwives, 20 (13%) nurses, and 30 (20%) auxiliary nurses/nursing assistants.^1^ Among the 150 providers surveyed, 110 (73%) were female, 92 (61%) were between 31 and 50 years old, 132 (88%) professed Islamic religion and 59 (39%) worked at a national hospital. More than half of the respondents (60%) had > 10 years of professional experience.

**Table 1:** Socio-demographic characteristics of health providers participating in the quantitative survey

	Midwives (N = 50)	Doctors		Nurses (N = 20)	Nursing Assistants/ATS (N = 30)	Total (%) (N = 150)
		Obstetricians/gynecologists (N = 15)	Other doctors (N = 35)			
*Age (years)*						
< = 30	11	1	4	1	12	29 (19)
31–40	17	3	11	5	9	45 (30)
41–50	15	8	12	7	5	47 (31)
> 50	7	3	8	7	4	29 (19)
*Sex*						
Male	1	12	24	0	4	41 (27)
Female	49	3	11	20	26	109 (73)
*Religion*						
Muslim	47	12	30	17	26	132 (88)
Christian	3	3	5	3	4	18 (12)
*Place of work*						
National hospital	14	11	20	9	5	59 (39)
District medical center	24	4	9	5	14	56 (37)
Health center	12	0	6	6	11	35 (23)
*Years of experience*						
< 1	4	0	0	0	0	4 (3)
1–2	4	0	1	0	3	8 (5)
2–3	3	1	0	0	2	6 (4)
3–5	4	1	2	0	1	8 (5)
5–10	7	2	13	3	9	34 (23)
10 +	28	11	19	17	15	90 (60)

ATS–Agent Technique en Sante: Auxiliary nurse/Nursing assistant cadre that complete three years of training in a professional health school after completing secondary school

A total of 37 IDIs were conducted among health care providers, including eight midwives and eight nurses (four each from an urban area (Conakry) and four each from a rural area; four doctors specializing in obstetrics and gynecology, who were sampled only from urban areas since there are few doctors practicing in rural areas. An additional eight IDIs were conducted among health service managers, directors of health faculties and schools, and representatives of health professional associations to gain an in-depth understanding of the health systems structure in responding to FGM and the codes of conduct regarding medicalization of FGM (Table 2).

**Table 2:** Socio-demographics characteristics of health providers and other key informants in qualitative interviews

In-depth interviews with health care providers				
	Midwives (N = 8)	Doctors (N = 4)	Nurses (N = 8)	TOTAL (%) (N = 20)
*Age (years)*				
Under 32	1	1	2	4 (20)
32–34	1	1	1	3 (15)
35–39	4	0	0	4 (20)
40 +	2	2	5	9 (45)
*Sex*				
Male	0	4	0	4 (20)
Female	8	0	8	16 (80)
*Location*				
Urban	4	4	4	12 (60)
Rural	4	0	4	8 (40)
*Religion*				
Muslim	4	4	7	15 (75)
Christian	4	0	1	5 (25)
*Years in the hospital/health center*				
< 2	3	2	3	8 (40)
2–5	1	1	1	3 (15)
6–10	2	0	2	4 (20)
10 +	2	1	2	5 (25)
*Years of experience*				
< 5	1	0	3	4 (20)
5–9	5	2	0	7 (35)
10–14	0	0	3	3 (15)
15–19	0	0	0	0 (0)
20–24	0	0	1	1 (5)
24 +	2	2	1	5 (25)
Total	8	4	8	20 (100)

In-depth interviews with other key informants				
	Health system managers (N = 6)	Directors of health professional associations (N = 3)	Directors of health schools (N = 4)	Total (%) N = 13)
*Age (years)*				
30–34	6	3	4	13 (100)
*Sex*				
Male	5	1	4	10 (77)
Female	1	2	0	3 (23)
*Location*				
Urban	4	3	3	10 (77)
Rural	2	0	1	3 (23)
*Religion*				
Muslim	6	3	2	11 (85)
Christian	0	0	2	2 (15)
*Years of experience*				
< 5	1	1	0	2 (15)
5–10	1	0	2	3 (23)
11–15	2	0	1	3 (23)
16–20	0	2	0	2 (15)
20 +	2	0	1	3 (23)

Another nine IDIs were conducted among health facility users receiving antenatal care who were predominantly 20–29 years old, out of school and married. A total of 11 FGDs were conducted separately among male and female community members. Women who participated in the FGDs were mostly less than 20 years old, married, out of school and not formally employed. Men who participated in the FGDs were predominantly less than 25 years old, unmarried, and out of school (Table 3).

**Table 3:** Socio-demographic characteristics of exit clients and community members participating in in IDIs and FGDs

	Female community members (N = 47)	Male community members (N = 37)	Exit clients (N = 9)	Total (%) (N = 93)
*Age (years)*				
< 20	12	10	1	23 (25)
20–24	10	9	3	22 (24)
25–29	5	4	3	12 (13)
30–34	1	1	0	2 (2)
35–39	6	2	1	9 (10)
40 +	13	11	1	25 (27)
*Marital status*				
Single	7	20	0	27 (29)
Married	36	16	9	61 (66)
Divorced	2	0	0	2 (2)
Widowed	2	1	0	3 (3)
*Location*				
Urban	24	24	5	53 (57)
Rural	23	13	4	40 (43)
*Religion*				
Muslim	46	36	9	91 (98)
Christian	1	1	0	2 (2)
*Educational level*				
Not in school	25	10	6	41 (44)
Primary	7	4	1	12 (13)
Secondary	13	14	0	27 (29)
Professional	1	1	0	2 (2)
University	1	8	2	11 (12)
*Occupation*				
Housewife	17	0	1	18 (19)
Dressmaker	9	0	3	12 (13)
Salesman/saleswoman	7	5	2	14 (15)
Pupil/student	4	8	0	12 (13)
Farmer	3	6	1	10 (11)_
University graduate not employed	0	4	1	5 (5)
Other	7	14	1	22 (24)
*Number of living children*				
0	17	22	1	40 (43)
1–2	10	5	7	22 (24)
3–4	6	3	0	9 (10)
5–7	11	4	1	16 (17)
8–10	3	3	0	6 (7)

**Fig. 1:**
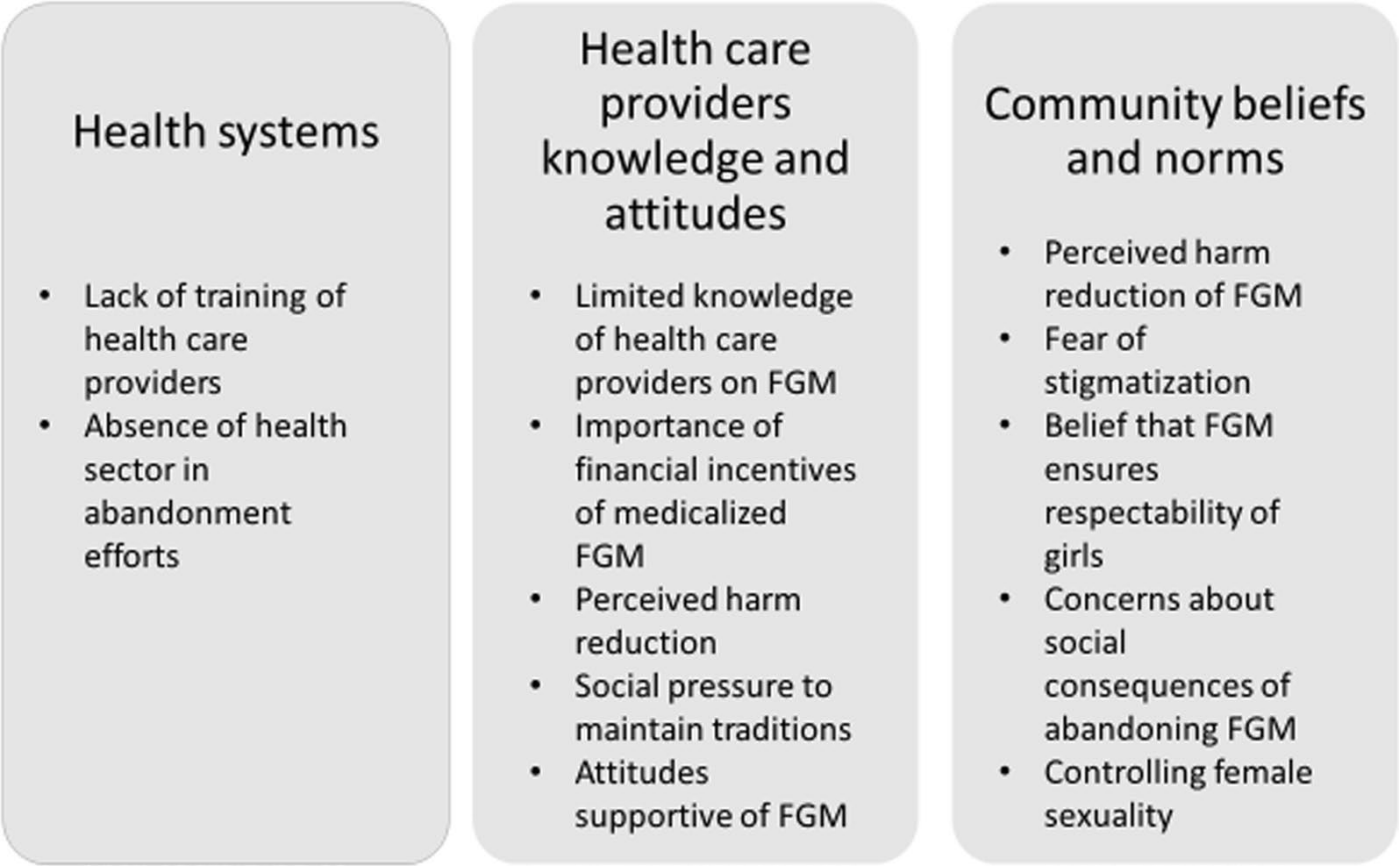
Factors that affect how the health sector provides FGM prevention and care services in Guinea

We present quantitative and qualitative findings using a conceptual framework that emerged during the data analysis, which includes three categories of factors that influence health care providers’ beliefs and decision-making about FGM, its medicalization and the care provided to women and girls at risk of or affected by FGM. Figure 1 presents the conceptual framework, which describes: (1) the factors intrinsic to the health system, which affect service provision; (2) the factors related to the health care provider herself/himself and (3) the community factors that drive demand and influence the provider, some of which have been described previously [11].

### Health systems factors that influence service provision

#### Lack of training

Twenty-four percent of all interviewed health care providers acknowledged that they did not have the requisite knowledge and skills to provide good quality care to women and girls who have undergone FGM (Table 4). This was especially true among medical doctors (45.7%). In Conakry, two thirds of the health care providers surveyed had not received any specific training on FGM during their basic and/or specialized training. This information was corroborated by individual IDIs. A nurse in Faranah-Marella reported: “When I was in school, I did not receive a special course on excision or genital mutilation … No, I didn't have any training on that, neither in treatment nor in genital mutilation.”

**Table 4:** Health providers’ knowledge regarding FGM

	Midwives	Doctors	Gynecologists	Nurses	Nursing Assistants/ATS*	Total (%) N = 150	
*How many types/categories of FGM do you know?*							
None	0	26	0	10	13	15	10
One	60	34	20	65	57	75	50
Two	24	23	20	25	23	35	23
Three	12	14	20	0	7	16	11
Four	4	3	33	0	0	8	5
Other	0	0	7	0	0	1	1
*When you receive a girl or a woman who has undergone FGM, do you think you have enough knowledge/skills to provide good quality care?*							
Yes	84	54	93	90	63	112	75
No	12	46	7	10	37	36	24
Don’t know	4	0	0	0	0	2	1
Total	100	100	100	100	100	150	100
*During your basic training and/or specialization, did you receive specific training on FGM?*							
Yes	52	11	33	25	33	50	33
No	48	89	67	75	67	100	67
Don’t know	0	0	0	0	0	0	0
Total	100	100	100	100	100	150	100

*ATS–Agent Technique en Sante: Auxiliary nurse/Nursing assistant cadre that complete three years of training in a professional health school after completing secondary school

According to a representative of professional organizations, most medical training establishments, including the Faculty of Medicine in Conakry, did not have specific modules on FGM. A school director in Labé reported: “No, in any case at this level and at the level of secondary health schools this program does not exist because I give some courses.” This low level of training on FGM related topics was further exemplified by the poor familiarity of health care providers on matters related to FGM. For example, 50% of the providers in Conakry knew only one FGM type (out of 4), 23% knew only two types, and nearly 10% of respondents did not know any type (Table 4). This low level of training among health care providers was also indicated by lack of knowledge on local regulations against FGM with 11% of respondents stating that FGM was a legal practice in Guinea although there has been a law prohibiting it since 2016.

#### Lack of involvement of health sector in abandonment efforts

According to health system managers, strategies, and programs to prevent FGM, mainly focused on awareness-raising carried out at the community level by donor/multi-lateral partner institutions and other non-governmental organizations, mainly led by the Ministry of Social Action. There was no FGM prevention program specific to the health sector. Rather, health sector managers are invited to workshops to launch these prevention activities and implement the resulting recommendations, as reported by this health system manager based in an urban area: “The last activity we carried out was to train all the midwives in the region to reduce or even eliminate female circumcision, in partnership with the Ministry of Social Action and supported by UNICEF.”

### Factors related to health care providers

#### Limited knowledge on management of complications

Another theme that emerged in the IDIs was the lack of capacity of health care providers to manage cases of infibulation (Type III FGM) due to limited knowledge on the practice in general and on how to perform deinfibulation. A doctor in Labé reported: “This procedure, which must be done on the day of childbirth if the baby does not come out normally, as is commonly said, is to increase a little, if necessary, under anesthesia, to make her understand that it is so that she does not feel pain, because there has been another act that has created problems at the level of the perineum previously, which is the one that has made her feel pain during sexual intercourse, and that it [deinfibulation]must be done during the childbirth.” From the quantitative survey, regarding the quality of care provided to women and girls who have undergone FGM, 41% of health care providers reported that they did not look for evidence of FGM during a routine gynecological examination, and only 5% of providers reported cases of FGM identified during their gynecological examination in the medical file or consultation register. In addition, 15% of respondents stated that they had never referred FGM-related complication to specialists.

##### Financial incentives to perform FGM

Among health care providers, we identified incentives to perform FGM as an important driver of FGM medicalization. A doctor from Labé said: “I have explained to you at the beginning, it is through these little cash gifts, and secondly, it is by ignorance, it is both. It's an economic problem that's there and a mental problem. It’s a problem of will, there are others who have received training, and despite all that, they take part in it.” This was the opinion of an exit client: “When will they stop excising? When they excise, they give medicine, we go home. You come back for the dressings until it's healed, once it's over. For their reward? Sometimes we give cereals, soap, and some money when it's over. Sometimes after you leave, you send them something. What they ask for is soap and cereal, which is compulsory, if you have the money, you will make them happy; if you don't have any, they take what you have after you’ve gone home.”

##### Perceived ‘harm reduction’ with medicalized FGM

FGM performed by health care providers was considered to reduce the risk of the complications associated with the practice. A 28-year-old midwife living in a rural area said: “because they think that if they send their girls to the providers for excision, there will be no complications such as bleeding and if there are complications, they know how to deal with them.” A doctor in a regional hospital reported the same: “Well, the first thing is why women might rely on health workers, especially the midwives. Well, send your daughters, we only remove a little, not the whole clitoris, we remove a little and leave the other one, so the orgasm will be and when the woman will grow up she will feel, that is the second thing, the second, here we think that in the health facilities there is a bit of hygiene, there is not too much risk of infection, that's all.”

Further, a health system manager and leader of professional health association interviewed shared the same opinion: “I already say that excision is certainly dangerous, it leads to death, it leads to hemorrhage, so we can avoid death and hemorrhage, but as we do not want to abandon the practice, we prefer to go to the health worker, where we think we're much safer, and that's certainly the argument we use to say yes, if you go to the hospital you're safe, if there's no hemorrhage there's no infection you can avoid tetanus because with AIDS there's been a lot of talk about the same knife eh.. the… or in any case the …the same instrument in several people leads to contamination the same unsterilized instrument in one person leads to infections and we say to ourselves well all that in hospital we can avoid…. We can avoid infection; we can avoid it.”

##### Social pressure to maintain the tradition

The practice of FGM was considered a cultural imperative. Not only did community members feel social pressure from elders and other family members and fear repercussions if their daughter failed to follow the family tradition prior to getting married, health care providers also felt pressure to maintain the tradition. As a result, community members request that it be performed by health professionals to avoid complications. For example, a nurse working in a rural area said: “Here I would have said it is to follow the custom, it is nothing but that.” This was also the opinion of an urban health sector manager: “It's a cultural issue, you know, in our cultures, our customs, it's always been a cultural problem, it's in our customs, so it's necessary to do it, people, for this reason, I think that even if in the past, maybe our great grandparents didn't know the seriousness of this.”

##### Support for the practice

As was described previously [11], health care providers themselves saw value in the practice as reported in their responses to a questionnaire on knowledge, attitudes, and practices. Thirty-eight percent of the respondents felt that FGM prevented promiscuity and infidelity, with 75% of nurses and 63% of auxiliary health providers supporting this belief.

### Factors related to community

#### Perceived harm reduction

The perceived harm reduction of medicalized FGM mentioned by health care providers was also brought out by community members. Female FGD participants in a rural area of Faranah and women in Conakry emphasized that: “The providers who do excision, for them it is good because when you go, they give you injections. Once they inject you now, they can do their work. For them it's better than the old women who excise children because with them (traditional cutters) there are no injections, nothing at all, they just take you and do it. The providers at least inject you to anaesthetize that part to tranquillize you until the end. For them it's better that the old ones do it, not in school, married.”

“We don't have any worries about that. Now, some people no longer practice excision in the traditional way, but practice it in hospitals because when the practice is done in hospitals the children bleed less. If the practice is done traditionally the children bleed a lot since the complete removal of the clitoris is done which makes the parents very anxious.”

A similar opinion was also shared by male FGD participants, such as this young man in Conakry who believed that the threat of complications was reduced if FGM was performed by a health care provider: “Doing the excision at hospital means that there is security, peace of mind because those who are there know their work, they are the ones who can do it in a way that gives you peace of mind. Since we are there all the girls who have had it done at the hospital have not had any problems.” Another male FGD participant said: “As my predecessors have just said, it is a custom and people continue to practice it. The fact that they are now carrying it out in hospital, we don't see any disadvantages because they know the equipment that should be used and their tools are disinfected, which can save us from infectious diseases. It's better than doing it in the villages, especially with different infectious diseases that we have now. We only take one knife and use it for two or three people. If we really must continue this practice, it is better to take them to hospital because they are health professionals.”

#### Fear of stigmatization

We found that girls who had not undergone the practice of FGM and their respective families were stigmatized by the community for non-conformity to this social norm. An adult woman in Conakry reported that such girls are rejected and mocked by their peers who have undergone FGM: “What we know about excision, you know, girl’s excision, before girls were excised without any problem, it didn't prevent them from having children, it's a custom that our grandparents practiced, we too we followed the same customs. We excise them, because if your neighbor excises her daughter and your daughter is not excised, her friends will make fun of her by saying that she is not excised. So, it is because of these misunderstandings that we excise our daughters.”

The majority of the interviewed women stated that they did not want to be the first to stop the practice of FGM because of the uncertainties around what would happen next and because of fear of stigmatization and social exclusion of their daughters, like this client who was interviewed when exiting the clinic: “You know how everyone says that it is a custom, so no one wants to stop excision. So, I say to myself that this is why others are still practicing excision.” This was also the opinion of health care providers such as this midwife from an urban area who reported that in the South of Guinea, girls who had not undergone FGM were not allowed to participate in village activities: “They are systematic. In any case, people identify the family by saying that their daughters are not excised, and you should not even take, get married there. At home, they don't want their daughters to go into the forest, it's called the sacred forest, ah, and if you don't do it when the old women want to come to the village, we who haven't done that, they'll tell us to go back into the houses, close the doors behind us until they've finished their ceremony, we don't go out, it is like that for us, so if you don't practice it, you'll be hidden all the time So that's how it is.”

#### Respectability

Respondents reported that FGM was “a way to protect the honor of women and girls” and that a girl or woman who had not undergone the practice will not be respected by her peers. As one patient explained during an exit interview: “As far as the importance of excision is concerned, everyone says it is for respectability. If the person (concerned) is not excised, it means that she is not respectable. That's how I understand the importance of excision. That's what I know.” Another young woman in a rural FGD, also stated: “we say here that if you are not excised it is a disgrace and a shame. If you see that she is not married, it means that men are afraid of her, all this brings shame.”

#### Community concerns about social consequences of abandoning FGM and gender roles

The idea of abandoning FGM fills some community members with anxiety and uncertainty, not only about the honor and behavior of their daughters, but also about how this abandonment would bring a significant change in the society. Women especially feared that their daughters would lose their morality with the abandonment of this practice. A young, single woman studying at a vocational school said: “Presently, (…), the way we see the atmosphere at the moment is that everyone knows how people behave in different communities. A lot of us fear that our daughters will get into debauchery because nobody wants that! That's why they are … they are subjected to FGM.” A married young woman: “In view of the current atmosphere, if you see the current behavior of girls on the streets, you will find it pitiful. Nobody wants her daughter's future to be like this. Even the excised girls, they are already indulging in debauchery and even more so if they are not excised. If we stopped excision, we don't know how non excised girls would behave on the street. This is why it is difficult for many people to stop the excision of little girls.”

#### Controlling sexuality and protecting honor

The practice of FGM was seen as a means of controlling the sexuality of women and girls to preserve the honor of the family. Indeed, men expressed concern about the sexual behavior of a girl/woman who had not undergone FGM, who they believed could have an insatiable sexual desire that could lead to adultery and make him feel that he was not able to satisfy his wife. This adult male living in a rural area in Faranah describes this concern about women’s sexuality: “We think that there are non-excised women who have an exaggerated sexual need. Yet a Muslim adult when you get married, you have to be able to satisfy your wife but if she, she is never satisfied with her husband, if we follow the whites and our leaders allow such a practice, it will cause us a lot of trouble. Because childbirth was not a problem in the past, neither for the woman nor for the children.”

“As far as excising our women is concerned, we have grown up with these customs here, but today we have learnt that white people say that excision causes problems to our women and girls and we don't understand, there are some who say that when girls are excised it decreases their pleasure, that is, the feeling that the man should feel from the woman and the woman should feel from the man will no longer be there, that it diminishes the woman's love (sensitivity) and that if we don't do this, the woman will never refuse a man for sex. There are also those who say that excised girls will suffer during childbirth. It is very common to hear this nowadays in our communities. Yet our mothers and great-grandmothers used to give birth without any difficulty, so whites just don't want us to keep our old [cherished] customs.”

Some male community members stated that FGM can reduce a girl's sexual drive: “The importance of female excision here is that it reduces the sexual needs of the woman because the excised woman needs men less (for sex) than the non-excised woman. The importance here is to reduce the sexual need.” Related to this, in this setting, FGM is practiced at a time when young girls are provided with advice to avoid perceived sexual immorality. A young woman from an urban area argued in an FGD: “If we stop excision, we don't know how the non-excised girls will behave on the street. Therefore, it is difficult for many people to stop excision.”

## Discussion

Despite significant efforts by the Guinean government with technical and financial support from its bi- and multilateral partners to address FGM, the practice remains prevalent, with indications of increasing involvement of health care providers. The present study identified several factors among the health care providers themselves, factors related to the health system and to the community at large that influence how health care providers deliver FGM prevention and care services and whether they themselves carry out FGM. Understanding these factors can help to develop interventions and programs to strengthen the health sector’s role in FGM prevention and care.

This study revealed gaps in FGM-related training of health care providers in Guinea. Several authors have reported lack of knowledge of FGM among health providers [6, 12–16]. In this study in Guinea, 10% of the health providers surveyed did not know any type of FGM. Additionally, this study revealed that despite the existence of laws prohibiting the practice of FGM, financial motivations and community pressure contribute significantly to medicalization of FGM in Guinea. This finding is in line with previous reports [12, 13, 17–19]. The present study also revealed gaps in the quality of FGM prevention and care services offered by health care providers in Guinea. Health care providers rarely checked for the presence of FGM during routine gynecological examination or asked about FGM during routine medical consultations. This finding is in line with previous reports that suggest that most health care providers rarely identify or ask users of health services about the status, severity and possible complications related to FGM [17].

Our study revealed low reporting of FGM-related complications, especially any resulting from FGM conducted by health care providers. In areas of high FGM prevalence, WHO recommends that health care providers assess for and systematically record FGM cases during routine consultation [19]. Further, the identified drivers on FGM medicalization included financial motivations, reducing FGM complications as well as buy in of health care providers into social norms of the community, which may conflict with their professional obligations of promoting FGM abandonment.

We identified several community-level factors that contribute to the practice and persistence of FGM in Guinea. Health facility users and the communities served by health care providers, have concerns regarding FGM abandonment fearing that it would stigmatize their daughters, that they would lose respectability, that society would be changed negatively because of increasing debauchery and that marital problems would arise if women’s sexual desire was not controlled. These fears drove families to have their daughters undergo FGM. Our study also found that the perpetuation of FGM in Guinea was related to community gender roles and expectations regarding women's sexuality. We found that girls in Guinea underwent FGM to prevent them from engaging in premarital sex, which is considered taboo. The idea that communities perform FGM to ensure the ‘respectability’ and limit the sexual desire of women has been reported in previous studies [13].

Several studies underscore the role of community social pressure as a key driver in understanding why health care providers perform FGM and/or re-infibulation [12, 13, 20]. Our study found that some health care providers held a positive attitude towards FGM, whether they had undergone FGM themselves or had maintained the tradition for their daughters, indicating that it might not always be clear how to distinguish between the personal beliefs and professional obligations of providers. The present study also found that the perceived ‘harm reduction’ argument was used to justify health care providers performing FGM in Guinea, as was reported in other studies [11–13, 15, 18, 21].

### Strengths and limitations

These results should be interpreted in the context of several limitations. First, we did not assess other building blocks of the health system, that may be create an enabling environment for the prevention and management of FGM. However, we did assess indicators of quality of health services provided and this information could be useful in making inferences at health system level about how to implement a stronger health sector response to FGM. Second, the fact that this research was conducted in three of the country's eight regions could have affected the generalizability of our findings. However, data collection took place in areas with sufficiently large numbers of the different cadres of health care providers and measures were taken to ensure the representativeness of the different cadres in Conakry. For the qualitative component, every effort was made to select enough participants of different characteristics to ensure that a range of perspectives was captured. Further research that involves other regions of Guinea or settings with similar characteristics with a larger sample size may be necessary to replicate our findings.

The main strength of this study is the identification of factors at the intersection of the health system and the community that contribute to the persistence of the practice of FGM and are amenable to interventions. In many settings like Guinea, health care providers are influential members of their communities and are subject to the same cultural norms and expectations. Our findings indicate that factors, such as holding attitudes supportive of FGM and financial/material incentives together with high community demand act in concert to perpetuate FGM medicalization. For this reason, interventions by the health sector would need to address factors at this intersection in developing comprehensive strategies to promote FGM prevention and care, and to prevent FGM medicalization. These could include enacting policy and regulations, developing both pre- and in-service FGM training content, including discussing attitudes that perpetuate the practice as well as implementing initiatives to counteract the incentives that health providers are offered to perform FGM.

### Implications for practice and research

Several areas will need to be addressed to strengthen the health sector’s role in FGM prevention and care. First, the health sector will need to have clear strategic areas of interventions to be implemented through health managers at the district level. Second, standardized training content adapted to the needs and skill sets of health care providers need to be developed and implemented. Third, it is important to address values and norms of health care providers themselves that conflict with the services they will provide on FGM prevention and care as well as address systemic issues regarding incentivization of health care providers to perform FGM. Finally, it is important for health care providers to recognize and address the values and fears of each individual and community they work with during either consultation visits or community health outreach activities. These interventions will need to be developed and tested or evaluated during implementation using research or monitoring and evaluation to ensure efficiency and effectiveness as part of multi-sectoral FGM abandonment efforts.

The results we present have informed the development of a health sector intervention, which is being field-tested in a multi-country, hybrid effectiveness-implementation cluster randomized trial, in collaboration with research institutions in three countries i.e., Guinea, Kenya and Somalia [8]. The intervention package applies a social norm change approach in which health care providers, acting as opinion leaders, transmit FGM prevention messages to their patients during routine or specialized health service provision. The findings from this multi-country study will expand the evidence base on what role the health sector can play as part of multi-sectoral FGM prevention efforts. Applying evidence-based approaches can help guide ministries of health and partners to enact interventions that build the knowledge and skills of health care providers on FGM prevention and care in a sustainable manner, using a health system strengthening approach.

## Conclusion

Our study highlights factors that could be targeted to strengthen the role of the health sector in the prevention of FGM and management of its complications. The lessons learnt from this formative research demonstrate the need to build the capacity of health care providers on FGM typology, management of complications and FGM-related principles of human rights and medical ethics. This study also highlights the need to engage health care providers around their own beliefs and values, which can influence the ways in which they provide care and the messages they transmit directly or indirectly about harmful practices such as FGM. Health system strengthening to improve FGM prevention and care services is also needed to ensure information and health policies reach providers in training or those working at the facility level.

## Data Availability

The study data set will be made available upon request.

## Abbreviations

CERREGUI: Cellule de recherche en santé de la reproduction en Guinée (Centre for Research in Reproductive Health in Guinea)
CSPro: Census and Survey Processing System
ERC: Ethical Review Committee
FGD: Focus group discussion
FGM: Female genital mutilation
IBM: International business machines
IDI: In-depth interview
SPSS: Statistical Package for the Social Sciences
WHO: World Health Organization

## Footnotes

1 Nursing assistants complete three years of training in a professional health school after completing secondary school.

## References

[1] Female genital mutilation/cutting: a global concern UNICEF. New York. 2016.

[2] Organization WH (2008). Eliminating female genital mutilation: an interagency statement-OHCHR, UNAIDS, UNDP, UNECA, UNESCO, UNFPA, UNHCR, UNICEF, UNIFEM.

[3] Who T (2016). WHO Guidelines on the Manajement of Health Complications from Female Genital Mutilation.

[4] Institut National de la Statistique, ICF. Guinea Demographic and Health Survey (EDS V) 2016–18. Conakry, Guinea: INS/Guinea and ICF; 2019.

[5] Organization WH. Global strategy to stop health-care providers from performing female genital mutilation. World Health Organization; 2010.

[6] Doucet M-H, Pallitto C, Groleau D (2017). Understanding the motivations of health-care providers in performing female genital mutilation: an integrative review of the literature. Reprod Health.

[7] Pallitto CC, Ahmed W (2021). The role of the health sector in contributing to the abandonment of female genital mutilation. Med.

[8] Ahmed W, Mochache V, Stein K, Ndavi P, Esho T, Balde MD (2021). A hybrid, effectiveness-implementation research study protocol targeting antenatal care providers to provide female genital mutilation prevention and care services in Guinea, Kenya and Somalia. BMC Health Serv Res.

[9] Chambers R (1994). The origins and practice of participatory rural appraisal. World Dev.

[10] Malcolm A, Aggleton P. Rapid assessment and response Adaptation guide for work with especially vulnerable young people. Institute of Education, University of London. 2004.

[11] Balde MD, O'Neill S, Sall AO, Balde MB, Soumah AM, Diallo B (2021). Attitudes of health care providers regarding female genital mutilation and its medicalization in Guinea. PLoS ONE.

[12] Berggren V, Salam GA, Bergström S, Johansson E, Edberg A-K (2004). An explorative study of Sudanese midwives’ motives, perceptions and experiences of re-infibulation after birth. Midwifery.

[13] Njue C, Askew I. Medicalization of female genital cutting among the Abagusii in Nyanza Province, Kenya. 2004.

[14] Dawson A, Homer CS, Turkmani S, Black K, Varol N (2015). A systematic review of doctors’ experiences and needs to support the care of women with female genital mutilation. Int J Gynecol Obstet.

[15] Mostafa SR, El Zeiny NA, Tayel SE, Moubarak EI (2006). What do medical students in Alexandria know about female genital mutilation?. East Mediterr Health J.

[16] Umar AS, Oche M (2014). Medicalization of female genital mutilation among professional health care workers in a referral hospital, north-western Nigeria. J Reprod Biol Health.

[17] Refaat A (2009). Medicalization of female genital cutting in Egypt. EMHJ East Mediterr Health J.

[18] Bjalkander O, Bergstrom S, Almroth L, Leigh B (2012). Female genital mutilation in Sierra Leone: who are the decision makers?. Afr J Reprod Health.

[19] Organization WH. Care of girls and women living with female genital mutilation: a clinical handbook. 2018.

[20] Shell-Duncan B, Gathara D, Moore Z. Female genital mutilation/cutting in Kenya: is change taking place? Descriptive statistics from four waves of demographic and health surveys. 2017.

[21] Ali AAA (2012). Knowledge and attitudes of female genital mutilation among midwives in Eastern Sudan. Reprod Health.

